# A Deep Learning Methodology for Screening New Natural Therapeutic Candidates for Pharmacological Cardioversion and Anticoagulation in the Treatment and Management of Atrial Fibrillation

**DOI:** 10.3390/biomedicines13061323

**Published:** 2025-05-28

**Authors:** Tim Dong, Rhys D. Llewellyn, Melanie Hezzell, Gianni D. Angelini

**Affiliations:** 1 Bristol Heart Institute, Translational Health Sciences, University of Bristol, Bristol BS2 8HW, UK; 2 Pharmacy Department, Liverpool Heart and Chest Hospital, Thomas Dr, Liverpool L14 3PE, UK; 3 Bristol Veterinary School, University of Bristol, Langford House, Langford, Bristol BS40 5DU, UK

**Keywords:** deep learning, bioinformatics, drug discovery, transcriptomics, proteomics, atrial fibrillation, machine learning

## Abstract

**Background:** The treatment and management of atrial fibrillation poses substantial complexity. A delicate balance in the trade-off between the minimising risk of stroke without increasing the risk of bleeding through anticoagulant optimisations. Natural compounds are often associated with low-toxicity effects, and their effects on atrial fibrillation have yet to be fully understood. Whilst deep learning (a subtype of machine learning that uses multiple layers of artificial neural networks) methods may be useful for drug compound interaction and discovery analysis, graphical processing units (GPUs) are expensive and often required for deep learning. Furthermore, in limited-resource settings, such as low- and middle-income countries, such technology may not be easily available. **Objectives:** This study aims to discover the presence of any new therapeutic candidates from a large set of natural compounds that may support the future treatment and management of atrial fibrillation anywhere using a low-cost technique. The objective is to develop a deep learning approach under a low-resource setting where suitable high-performance NVIDIA graphics processing units (GPUs) are not available and to apply to atrial fibrillation as a case study. **Methods**: The primary training dataset is the MINER-DTI dataset from the BIOSNAP collection. It includes 13,741 DTI pairs from DrugBank, 4510 drug compounds, and 2181 protein targets. Deep cross-modal attention modelling was developed and applied. The Database of Useful Decoys (DUD-E) was used to fine-tune the model using contrastive learning. This application and evaluation of the model were performed on the natural compound NPASS 2018 dataset as well as a dataset curated by a clinical pharmacist and a clinical scientist. **Results:** the new model showed good performance when compared to existing state-of-the-art approaches under low-resource settings in both the validation set (PR AUC: 0.8118 vs. 0.7154) and test set (PR AUC: 0.8134 vs. 0.7206). Tenascin-C (TNC; NPC306696) and deferoxamine (NPC262615) were identified as strong natural compound interactors of the arrhythmogenic targets ADRB1 and HCN1, respectively. A strong natural compound interactor of the bleeding-related target Factor X was also identified as sequoiaflavone (NPC194593). **Conclusions:** This study presented a new high-performing model under low-resource settings that identified new natural therapeutic candidates for pharmacological cardioversion and anticoagulation.

## 1. Introduction

Existing bleeding risk scores are notorious for poor prognostic capabilities. More recently, studies have examined the potential of using novel biomarkers for bleeding risk prognosis [1]. This is important not only for guiding oral anticoagulant administration in terms of dose for atrial fibrillation (AF) patients but also for preventing stroke since risk factors for bleeding and stroke often overlap. The patient subgroup of key importance is the low stroke risk group (CHA_2_DS_2_-VASc score 1), for which the appropriate anticoagulant dosage is most difficult to determine [1]. Overestimation of bleeding risk leads to anticoagulants not being sufficiently used, leading to a higher proportion of mortality and morbidity in AF patients, including stroke, which could have been avoided otherwise [2]. A study by Siegbahn et al. applied a Proximity Extension Assay to identify key biomarkers differentially regulated in bleeding: growth differentiation factor-15 (GDF-15), high-sensitivity cardiac troponin T (cTnT-hs), and seven novel biomarkers: osteopontin, ephrin type-B receptor 4, tumour necrosis factor (TNF) receptor 1, TNF receptor 2, soluble urokinase plasminogen activator receptor, TNF-related apoptosis-inducing ligand receptor 2, and osteoprotegerin from a large set of 268 unique protein biomarkers in plasma samples [3]. The increased levels of cytokine GDF-15 have been shown to be associated with cellular stress, tissue damage, and heightened risk of bleeding in AF patients. This was also shown in various other disease groups such as cerebral haemorrhage, acute coronary syndrome, pulmonary embolism, etc. [4]. However, the limitation of GDF-15 [5] and other biomarkers such as neutrophil-to-lymphocyte ratio (NLR) [6] is that it modifies the risk of not only bleeding but also a range of other cardiovascular and non-cardiovascular outcomes, including stroke, mortality, heart failure, and cancer [4]. Newer scores need to focus on biomarkers with simpler, more specific (direct) mechanisms to be incorporated into antithrombotic therapy guidelines.

The ABC bleeding risk score is an existing score of the risk of bleeding in AF patients and consists of two clinical risk factors (age and history of bleeding) and three biomarkers (GDF-15 (a marker of oxidative stress), cTnT-hs (marker of myocardial injury), and haemoglobin) [7]. This ABC score outperformed two other bleeding risk scores, HAS-BLED and ORBIT, achieving a c-index of 0.71 in the RE-LY external validated trial [7]. In terms of the use of artificial intelligence (AI) in the management of AF, studies have focused mainly on the use of AI for screening of AF rather than other aspects such as biomarker discovery [8,9].

### 1.1. Current Clinical Drug Practice for AF

NICE British National Formulary (BNF) recommends that cardioselective drugs (specifically affecting receptors in the heart) such as bisoprolol be used for AF treatment [10]. These drugs help reduce unwanted systemic effects by targeting cardiac cells. Alternative antiarrhythmic drugs include the potassium and β-receptor blocker sotalol, which does not interact with warfarin, although it is reported to have minor interactions with aspirin [11]. Flecainide, a sodium channel blocker is also used and does not appear to have any significant interactions with anticoagulant medications. Digoxin, is indicated for maintenance of atrial fibrillation or flutter and is used mostly to slow atrioventricular nodal conduction through parasympathomimetic effects, thereby decreasing the ventricular response rate and reducing the impact of the irregular rhythm. Digoxin may exacerbate the risk of bradycardia if co-administered with beta-blockers [11].

### 1.2. Anticoagulants for Managing Risk of Clotting in AF Patients

Whilst anticoagulants help to manage the risk of stroke and blood clot formation in patients with AF, these increase the risk of bleeding. Therefore, a fine balance needs to be achieved. Warfarin is a vitamin K antagonist anticoagulant and strongly affects anticoagulant interaction pathways [12]. One limitation of warfarin is that there needs to be continuous monitoring of dosage and safety due to its interaction with other drugs and food, as well as the variable natural response to warfarin treatment and impact on International Normalised Ratio (INR) [13]. Direct-acting oral anticoagulants (DOAC) such as rivaroxaban [14] and apixaban, both factor Xa inhibitors, are approved by NICE for the management of clotting risks in humans [15] and are increasingly preferred over warfarin, except for patients with recorded allergies or metallic heart valves. They avoid the need for routine laboratory monitoring and dosage adjustments. Whilst clopidogrel is used as the primary drug for preventing thrombotic events in veterinary cardiac settings (most commonly in cats), clopidogrel is considered a second-line treatment inpatients who cannot take warfarin [16].

### 1.3. Aim

This study aims to discover the presence of any new therapeutic candidates from a large set of natural compounds that may support the future treatment and management of atrial fibrillation using a low-cost technique. Graphics processing units (GPUs) are expensive and often required for deep learning. However, in low-resource settings, for example, such as low- and middle-income countries, such resources may not be a commodity [17]. The objectives were to develop a deep learning approach to apply to atrial fibrillation as a case study using a low-cost technique where suitable high-performance NVIDIA graphics processing units (GPUs) are not required and to identify candidate therapeutic compounds that could be used everywhere.

## 2. Methods

### 2.1. Dataset and Materials

The primary training dataset is the MINER drug–target interaction (DTI) dataset from the BIOSNAP collection (Table 1) [18]. It includes 13,741 DTI pairs from DrugBank, 4510 drug compounds, and 2181 protein targets. The Database of Useful Decoys (DUD-E) is used to fine-tune the model using contrastive learning. The DUD-E dataset analysed consisted of 57 protein targets and active compounds that are known to interact with these targets as well as decoys that are known not to bind the targets but have very similar chemical structures [19].

This evaluation of the model was performed on the Natural Product Activity & Species Source (NPASS) 2018 dataset as part of the case study (Table 2) as well as a dataset curated by a clinical pharmacist and a clinical scientist. The selection of clinical compounds and targets was based on the existing guidelines; the British National Formulary (BNF); commonly used compounds in the United Kingdom healthcare system; clinical expertise/prioritisation from the clinical pharmacist, cardiologist, and cardiac surgeon; as well as previous review work conducted, focusing on relevance for the treatment and management of AF [20]. The dataset curated by the clinical pharmacist consisted of (i) compound dataset: 12 antiarrhythmic drugs and 6 anticoagulant drugs commonly used in hospitals in the United Kingdom; (ii) target dataset: the target dataset consisted of representative targets whose genes are typically up-regulated in atrial fibrillation, consisting of beta-1 adrenergic receptor (ADRB1 gene) and potassium/sodium hyperpolarisation-activated cyclic nucleotide-gated channel 1 (HCN1 gene [20]) for antiarrhythmic drug-related targets and Factor Xa as the anticoagulation target. The antiarrhythmic drugs for AF were further divided into four main classes as well as other miscellaneous classes to visualise any relationships. Further subclassifications are beyond the scope of this work.

### 2.2. Target Featurisation

The chemical compounds were transformed into features using the Morgan fingerprint method. The protein sequences were transformed into features using the Skipgram Neural Network embedding approach as described by ProtVec [21]. The rationale for using this embedding approach over that of ProtBERT as in [22] is that the latter requires CUDA (NVIDIA GPU) to be available and configured to the appropriate version of the torch library on the device, which is not always available as is the case in this study. In addition, ProtBERT requires enormous computational power and requires continuous rapid connectivity to platforms such as Hugging Face, which may also pose challenges.

### 2.3. Modelling Approach

**Figure 1:**
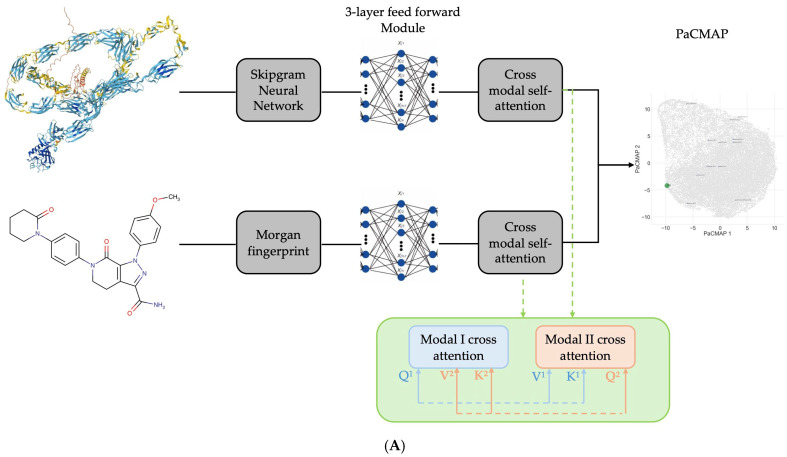

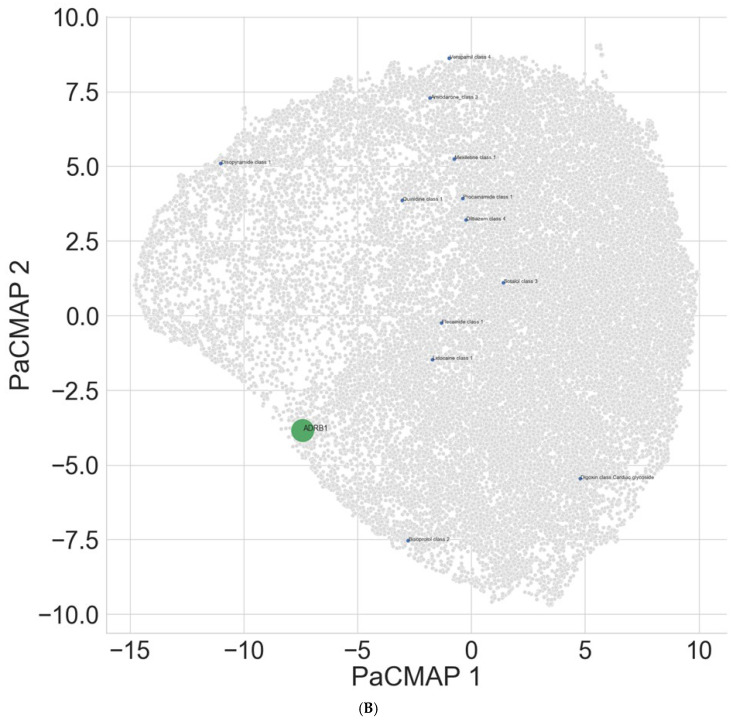
(**A**) A schematic overview of the modelling approach. (**B**) Using PaCMAP, the learned latent space for ADRB1 (green), commonly used clinical antiarrhythmics (blue), and natural compounds (grey) are shown.

A Schematic overview of the approach is provided in Figure 1A below.

The embeddings $E_{i}$ = $f_{i}\left(X_{i}\right)$ of the target (*i* = 1) and compound (*i* = 2) are entered separately into 3 layer feed-forward projection modules:

$$L_{1}^{i}=ReLU(W_{1}^{(i)T}E_{i}+b_{1}^{i})$$

(1) $$L_{2}^{i}=ReLU(W_{2}^{(i)T}L_{1}+b_{2}^{i})$$

$$L_{3}^{i}=ReLU(W_{3}^{(i)T}L_{2}+b_{3}^{i})$$

where *W* represents the weights and *b* represents the bias in each layer. Subsequently, a dual-headed cross-modal self-attention component is added:

(2) $$A=\left[\sigma \left(\frac{Q^{1}K^{2}}{\surd (p/2)}\right)V^{2},\;\sigma \left(\frac{Q^{2}K^{1}}{\surd (p/2)}\right)V^{1}\right]\;W_{G}$$

where p=1024, $W_{G}$ is the weight capturing the global contextual information across attention heads, $Q^{1}$ is the query matrix calculated using the target embedding, and $K^{2}$ and $V^{2}$ are calculated using the drug compound embeddings in the first attention head. In the second head, this is reversed, where $Q^{2}$ is the query matrix calculated using the drug compound embedding, $K^{1}$ and $V^{1}$ are calculated using the target embeddings. This composition is used to maximise both the local and global information captured across the two modalities.

Subsequently, residuals were added and layer normalisation was performed to propagate gradient and normalise features. Finally, a single-layer feed-forward linear classifier is added to conduct the classification. Binary cross entropy (BCE) loss with the sigmoid function was used for the optimisation process rather than standard BCE loss to ensure values are normalised strictly between 0 and 1 during this process.

(3) $$W_{4}^{T}\left(\frac{\hat{A}-\mu_{\hat{A}}}{\sqrt{\sigma_{\hat{A}}^{2}+\varepsilon }}\;\alpha +\beta \right)+b_{4}$$

where $\hat{A}=E_{1}+E_{2}+A,\;{\;\mu }_{\hat{A}}$ is the empirical average of $\hat{A}$, $\sigma_{\hat{A}}^{2}$ is the variance of $\hat{A}$, and α and $W_{4}^{T}$ are the weights of the normalisation and classification layers, respectively. β and $b_{4}$ are the bias of the normalisation and classification layers, respectively. ε is a small constant in the denominator that reduces the likelihood of numerical instability.

The above modelling process was applied to the BIOSNAP as initial pre-training. This was then followed by contrastive learning as per [22] using the DUD-E training dataset. The Triplet (contrastive) loss function was used in order to perform the contrastive learning component of the training process. For each drug compound interaction pair, 50 non-interacting decoys were sampled randomly to produce the triplets, and the loss values were averaged across each triplet set. To explain in more detail, the DUD-E dataset of positive samples with random samples of negative sample combinations whilst used to facilitate the contrastive learning were only applied to the MINER-DTI training dataset such that it aims to enhance the contrast between the MINER-DTI training dataset’s positive and negative sample pairs for drug–target interactions, whilst maximising the likelihood of interaction between the active compound in the DUD-E dataset and the target in the MINER-DTI training dataset. In this sense, the DUD-E’s decoy non-active samples act like an augmentation to the negative samples in the MINER-DTI training dataset.

### 2.4. Representation Approach

Due to projection to the latent space resulting in negative embedding values, which can have arbitrary spatial meanings in terms of its negativity (i.e., the negative spatial orientation is not an indication of dissimilarity by itself), the cosine similarity was not used, but Euclidean distance was used instead, which cannot be negative. Due to the curse of dimensionality, Euclidean distances may not be robust in directly calculating the distance in non-linear distances across high dimensionality data functions; hence, the PaCMAP approach was applied to preserve global and local structure of data in lower dimensional space (i.e., two-dimensional projection space) before calculating the Euclidean distance [23]. This distance metric is then a measure of the predicted extent of interaction between targets and compounds with smaller distances representing higher interactions. The model embeddings of the target, active, and natural compounds were projected and distances calculated using this approach. Depending on the target, the active compounds were changed to either antiarrhythmics or anticoagulants.

### 2.5. Model Evaluation

The new model was benchmarked against the non-contrastive model in [22], whereby the only difference is that the ProtBERT featurisation of targets is replaced with using Skipgram Neural Network embedding instead. As the process is still very computationally costly and the dataset is large, training, validation, and test split evaluation methodology was used instead of cross-validation [24]. Precision–Recall Area Under the Curve (PR AUC) was used to evaluate the performance of models on the validation and test datasets. Both models with and without contrastive learning were evaluated. However, since only the overall precision–recall performance is of interest in this study, as it does not concern with diagnostics and risk prediction specifically, the precision curve plot is beyond the scope of the current study. Due to the computational cost required to run the contrastive learning model, consideration of analysis using any evaluation metrics (including uncertainty quantification) are beyond the scope of the current study.

Python version 3.9 and torch version 2.1.2 were used for the analysis.

## 3. Results

The ConPLex achieved a satisfactory performance of 0.7154 and 0.7206 on the validation and test dataset, respectively (Table 3). The New model demonstrated a higher magnitude of performance in both the validation (0.8140 vs. 0.7154) and test set (0.8369 vs. 0.7206).

Using the highest-performing model from the above analysis, both the ConPLex and New models were supplemented with contrastive learning. Whilst the regularisation effect decreased the performance of the model slightly compared to its non-contrastive counterpart, it still outperformed the ConPLex model without contrastive learning in both the validation set (0.8118 vs. 0.7154; Table 4) and test set (0.8134 vs. 0.7206). In addition, it also outperformed the ConPLex model with contrastive learning in both the validation set (0.8118 vs. 0.6999; Table 4) and test set (0.8134 vs. 0.6943).

Table 5 shows that as expected, ADRB1 interacts mostly strongly with bisoprolol. In the projection space (Figure 1B), it can be seen that Sotalol is also closely positioned to the target compared to many of the other antiarrhythmics. Lidocaine and flecainide were found to interact with ADRB1, although these are likely to be interactions through closely associated mechanisms rather than direct binding.

Interestingly, class I drugs can be seen to form two clusters, with one cluster being lidocaine and flecainide and the other cluster composed of quinidine, procainamide, and mexiletine, suggesting potential similarities in interaction mechanisms.

**Figure 2:**
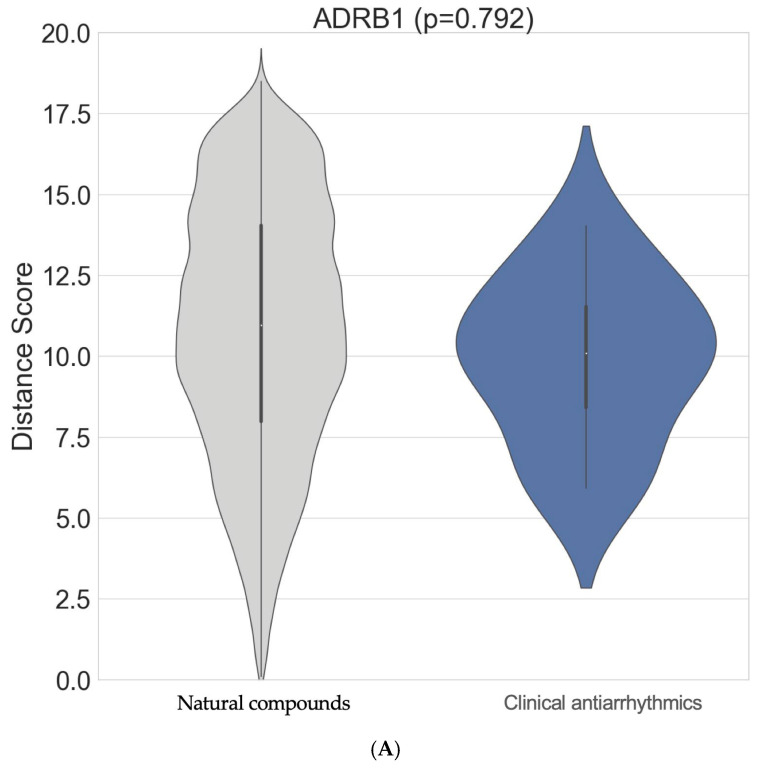

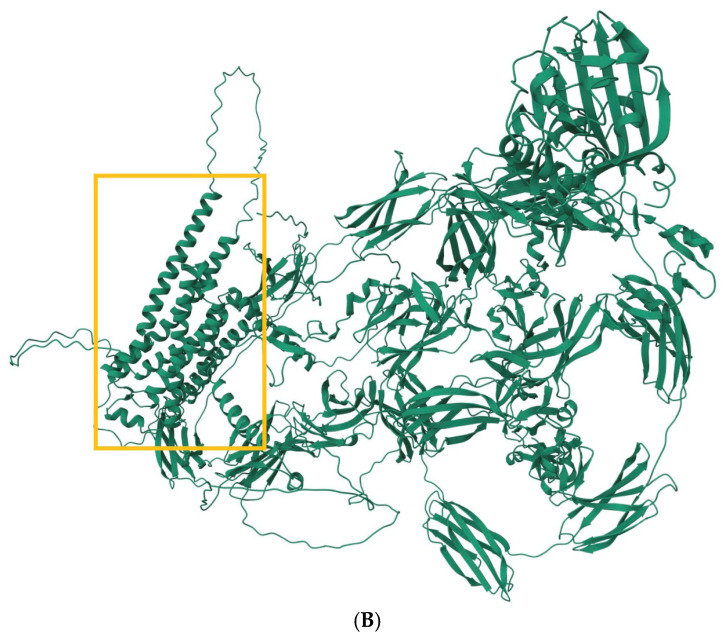
(**A**): Using a violin plot, the distribution of interaction scores for ADRB1 against the clinical antiarrhythmics (blue) and natural compounds (grey) are shown; *p*-value shows results from one-sided *t* test; (**B**): predicted 3D complex of ADRB1 (orange boxed region) bound to tenascin-C.

Although the top interactors for ADRB1 are not well known in the existing literature, tenascin-C (TNC; NPC306696) was identified as a strong natural compound interactor of ADRB1 (Figure 2B; Supplementary Materials Table S1). An ITScore-PP score of −280.142 using the MDockPP tool was obtained as a surrogate of a very high binding affinity for these compounds in this arrangement. The natural compounds did not interact with the ADRB1 target more strongly overall (*p* = 0.792). However, the tail of the grey distribution shows that some natural compounds demonstrated stronger binding scores than those of commonly used clinical antiarrhythmic drugs (Figure 2A).

As expected, lidocaine showed strong interaction with the HCN1 protein (Supplementary Materials Table S2). Interestingly, deferoxamine (NPC262615) was identified as the natural compound that interacts most strongly with the HCN1 protein (Supplementary Materials Table S3).

**Figure 3:**
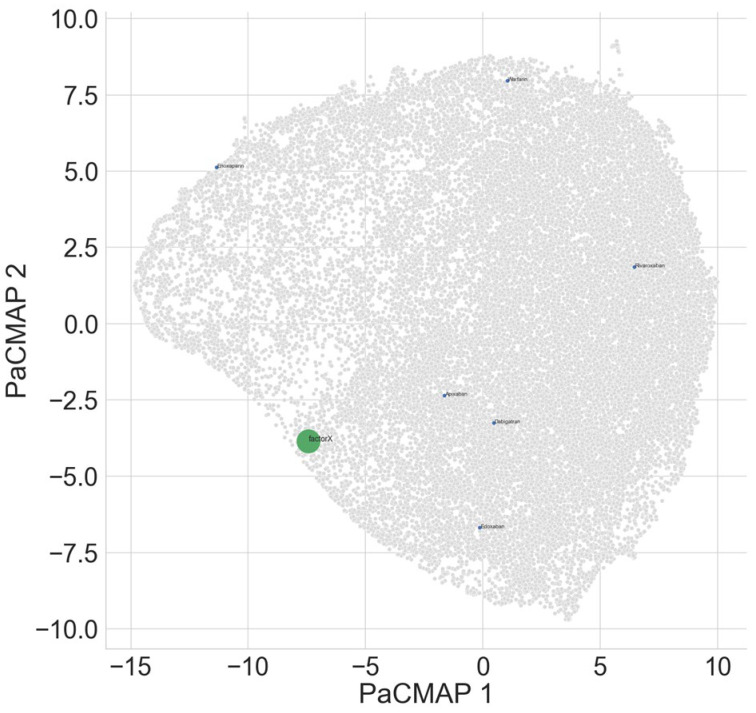
Using PaCMAP, the learned latent space for Factor Xa (green), commonly used clinical anticoagulants (blue), and natural compounds (grey) are shown.

Factor Xa was found to bind most strongly with apixaban as expected (Table 6). Warfarin showed less intense binding in contrast (Figure 3).

The third-strongest natural compound interactor of Factor Xa was identified as sequoiaflavone (NPC194593; Table 7). Figure S1 shows that the attention matrix for each of the cross-modal attention heads captures different sparse sets of information and hence may complement each other in terms of the decision-making process. The natural compounds did not interact with the Factor Xa target more strongly overall (*p* = 0.658). However, the tail of the grey distribution shows that some natural compounds demonstrated stronger binding scores than those of commonly used clinical anticoagulants, though this difference is marginal (Figure S2).

Supplementary Materials Table S4 shows that both the new model and the ConPLex model using the approach described here outperformed the computational time and hardware cost of the approach described in Singh et al. (ConPLex with GPU with ProtBERT featurisation) [22].

Whilst the PR AUC metric used earlier is particularly suited in scenarios of class imbalance, focusing only on the sample pairs with positive class for interaction, the BIOSNAP validation and test dataset were relatively balanced in terms of interaction pairs/number of non-interaction pairs (1396/1352 and 2770/2727, respectively). Hence, the Area Under the Receiver Operating Characteristic Curve (ROC AUC) performance metric, which is suitable for use under a balanced class distribution, was also assessed as a sensitivity analysis to further validate the reliability of the approach. Using this metric, the performance obtained showed a similar relationship to that of the PR AUC, with the new model outperforming the ConPLex model under both scenarios with and without contrastive learning (Supplementary Materials Tables S5 and S6).

**Figure 4:**
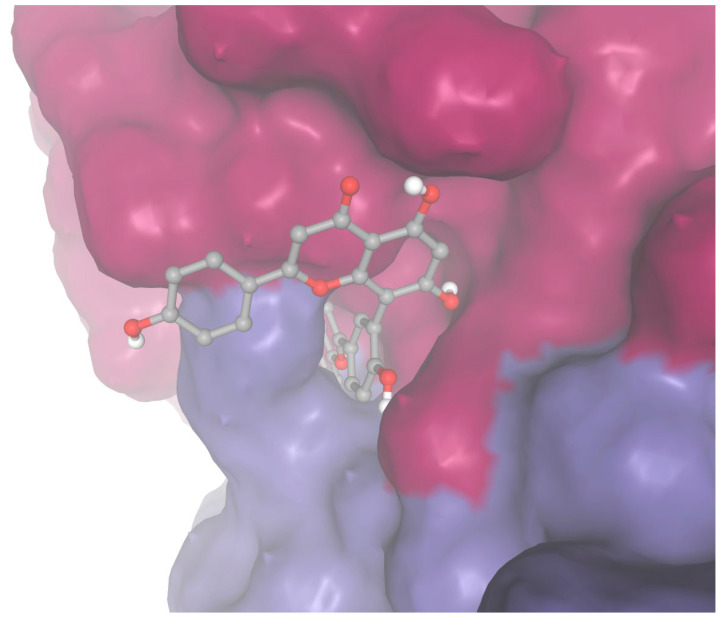
Docking of sequoiaflavone within the archway between the intersection between the heavy and light chains of Factor X.

As a sensitivity analysis, docking was performed for sequoiaflavone and apixaban against Factor Xa using Autodock Vina. Sequoiaflavone demonstrated a slightly higher binding strength for Factor Xa compared to apixaban (ΔG −5.59 vs. −5.569 kcal/mol; Figure 4).

## 4. Discussion

Existing work in DTI prediction has focused on areas beyond atrial fibrillation and there has been limited consideration of the use of natural compounds and dataset curated by clinical pharmacist in the analytical process for this specific purpose. For example, Wong et al. have developed a link prediction (predicting interactions with a network) method using Gaussian kernel-based network similarity matrices of miRNA and lncRNA to feed into a linear optimisation algorithm for predicting their interactions [25]. In addition, Wang et al. developed an innovative approach by extracting Graph attention network attention weights for input into an optimised deep learning algorithm, called the ensemble deep RVFL network (edRVFL), for learning from intermediate feature forms rather than high-level features [26]. The approach was used to effectively fuse heterogeneous multi-disease phenotypes with circular RNA (cRNA) to predict their interactions. Other DTI prediction studies have focused on techniques requiring the use of CUDA (NVIDIA GPU) to be available and configured to the appropriate version of the torch library on the device, which is not always available, as is the case in this study, or may be limited in terms of model explainability in the latent space [27,28]. This study aims to bridge this gap, focusing not only on the development of new improved modelling approaches but also the application of such model for drug target interaction prediction and therapeutic compound discovery.

**Figure 5:**
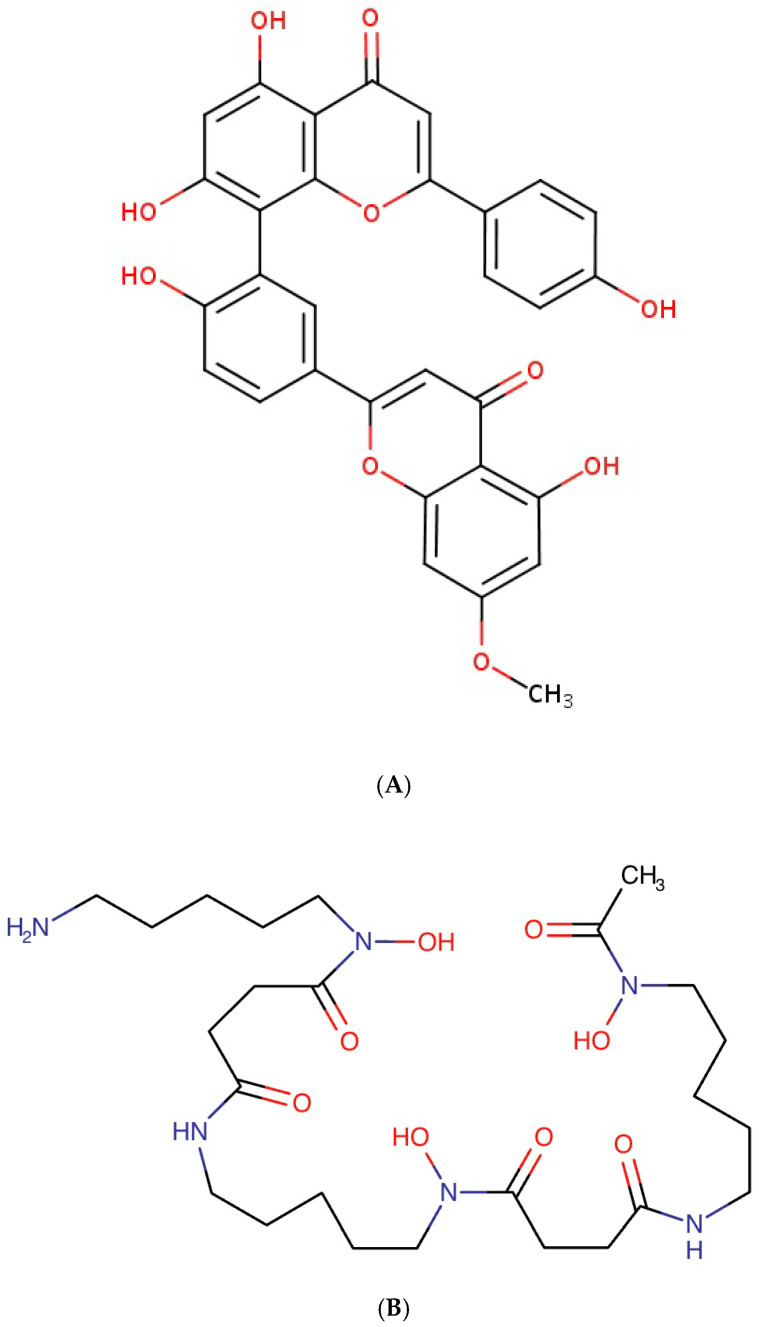
Chemical structure of (**A**): sequoiaflavone (PubChem) and (**B**): deferoxamine (DrugBank).

This study identified sequoiaflavone as a potential anticoagulant candidate for targeting Factor Xa. Sequoiaflavone is one of the five flavonoids present in the ginkgo biloba plant native to East Asia and may have anti-inflammatory [29] and cardioprotective effects through the inhibition of phosphodiesterases (PDEs) as well as anticancer effects through down-regulation of the PI3K/AKT signalling pathway [30]. The presence of phenolic hydrogens allows for these molecules to be donated to support the scavenging of reactive oxidative species (ROS) produced during inflammation (Figure 5A). The inhibition of PDE by sequoiaflavone could also decrease the hydrolysis of cyclic nucleotides [31] Cyclic adenosine 3′,5′-monophosphate (cAMP) and cyclic guanosine 3′,5′-monophosphate (cGMP), required for platelet aggregation [32]. It is interesting to note that in a previous study, sequoiaflavone was included as an ingredient in the ginkgo biloba extract (GBE50) that enhanced the antiplatelet effects of aspirin synergistically, reducing the effects of platelet aggregation [33]. Further clinical trials would be required to assess the independent effectiveness of sequoiaflavone in vivo for managing the risk of stroke and blood clot formation in animals and patients with AF, whilst minimizing the risk of bleeding. However, the parent drug Bio-Biloba—also containing the other flavonoids using ginkgo biloba, available in film-coated tablet form—is already approved by Medicines and Healthcare products Regulatory Agency (MHRA) and can readily be assessed further in clinical trials.

Deferoxamine is an iron chelator traditionally used to treat iron overload, transfusion-dependent anaemias, and chronic kidney disease (CKD)-related aluminium toxicity [34]. In parallel, it has been shown that increased accumulation of intra-cellular iron levels activates ferroptosis (produces ROS) and the Fenton reaction [34] (produces hydroxyl radicals) can lead to arrhythmia (including AF) development through ROS-induced ion channel remodelling, myocardial fibrosis, and mitochondrial dysfunction [35]. Previous studies have also shown that mycolactone, a toxin produced in bacteria, results in the hyperpolarisation of dorsal root ganglion (DRG) neurons [36]. In parallel, studies have shown that thoracic DRG depolarisation reduced ventricular arrhythmogenicity [37]. Supporting the results of this study, it was demonstrated previously that deferoxamine inhibited mycolactone-mediated cytotoxicity [38] and hence the associated understimulation through hyperpolarisation of DRG. Since deferoxamine is already approved for clinical use in the United States and other countries such as the United Kingdom (Figure 5B), it should be possible to conduct further clinical trials potentially repurposing these for in vivo testing of efficacy for the treatment of cardiac arrhythmia. Deferoxamine is typically administered through subcutaneous or intra-muscular injections. Hence, the drug is typically combined with the mesylate anion to increase solubility and hygroscopicity. A similar iron chelator that can be administered orally is also available, i.e., deferasirox, although such research has mainly focused on cancer treatment so far [39].

Interestingly, tenascin-C (TNC; NPC306696) was identified as a strong natural compound interactor of ADRB1. TNC is a large extracellular matrix glycoprotein characterised as a matricellular protein that is highly expressed during healthy and pathological tissue remodelling [40]. In particular, it can exert both harmful (proinflammatory and profibrotic effects) and beneficial effects in damaged hearts depending on its surrounding signalling factors. In particular, it is able to bind to more than 25 different proteins, including platelet-derived growth factor (PDGF), and hence have a wide range of functions, including oligomerisation, induction of mitogenic responses, cell migration, cell attachment, cell spreading, focal adhesion, cell survival, matrix assembly, and protease and proinflammatory cytokine synthesis [41]. TNC serum level has also been suggested as a prognostic marker of cardiac disease due to its inflammatory-related effects for a wide range of heart diseases including AF [42]. In terms of mechanism, it has been suggested that it works together with proinflammatory cytokines such as Interleukin-6 in a positive feed-back loop to enhance inflammation and promote myocardial fibrosis [42]. In addition, is has been found that in AF patients, the amount of TNC is correlated with the severity of atrial dilation [42]. While studies to date have mainly focused on targeting this TNC’s FNIII domain using antibodies and antagonists in cancer treatments [41], future therapeutic targeting to lower TNC levels could provide potential effective treatments for AF, but further evaluation would be required [43].

This study supported the results of previous studies that showed ADRB1 is inhibited by lidocaine with pH-associated effects on binding [44]. Flecainide was also identified previously as strongly interacting for ADRB1, with its potency effect for treating atrial fibrillation varying based on different genotypes [45]. However, the effect is indirect through the ADRB1 activation that facilitates the augmented inhibition of flecainide on sodium channels. NICE British National Formulary (BNF) recommends that cardioselective drugs (specifically affecting channels in the heart) such as bisoprolol (identified as the strongest-interacting drug for ADRB1) require less frequent dosing, since their duration of action is longer [10]. Sotalol, a non-cardioselective drug, was found to interact less strongly in this study, though different variants may interact differently [46]. In addition, sotalol is mostly used for its potassium channel blocking effects and hence may have comparatively lower effects on ADRB1. However, potassium channels were excluded from this study since potassium channels typically have decreased expression in atrial fibrillation patients [20]. Hence, further blockade of these channels may not always be beneficial. These drugs can be used in conjugation with digoxin (though used less nowadays) to control the ventricular response in atrial fibrillation [10].

Amiodarone, for example, is approved for the treatment of AF in both UK and European guidelines where other drugs are either not efficacious or are contra-indicated [47]. In this study, it was identified as a drug with low interaction effects to atrial fibrillation targets, potentially explaining why it might be helpful in scenarios, e.g., where drug interactions could lead to contra-indication. Amiodarone is an antiarrhythmic drug, classified as a potassium channel blocker, although it also has sodium-, calcium-, and β-receptor-blocking activity. Amiodarone is used to chemically cardiovert AF patients to a normal sinus rhythm. In terms of interactions with anticoagulant drugs, amiodarone increases the effect of anticoagulants such as warfarin through the inhibition of coumarin [12].

Surprisingly, despite rivaroxaban being approved by NICE, it was shown to demonstrate lower interaction with Factor X than warfarin, which is now decreasingly used. Nonetheless, clinical trials showed that rivaroxaban actually increased the risk of major bleeding compared to the use of blood-thinning aspirin only group in coronary artery and peripheral artery disease patients [48]. One additional limitation of these novel oral anticoagulants (NOACs) alone with dabigatran is that whilst they do not require dosage monitoring as with warfarin, they may have decreased persistence in usage by patients, leading to worse clinical outcomes.

Previous studies using the DUD-E dataset have been affected by limitations in the DUDE dataset in terms of limited chemical space, analogue bias, and decoy selection bias [49]. The decoys are small molecules that are known to not bind the target yet share physicochemical characteristics with the actual interacting compound for each target, and this could have a selective bias if manual selection of a subset of decoys is used for inclusion should that selection be carried out in a systematic manner. However, here, the random nature of the decoy sampling process mitigates such bias. Unlike other studies such as that in Chen et al. [49], which considers the DUD-E dataset as the sole dataset for training and test evaluation, the DUD-E dataset here is used to support the training process conducted on the MINER-DTI BIOSNAP dataset through contrastive learning, reducing any likelihood of restrictions to the chemical space exploration by the negative samples of non-interacting drug–target pairs.

In this specific study, there were no overestimations observed as a result of using the DUD-E dataset contrary to other studies [49], perhaps due to the difference in methodology of contrastive learning applied here. Instead, the use the of DUD-E dataset with contrastive learning showed a slight regularisation-like effect where the prediction performance decreased slightly in the trade-off to prevent overfitting. Future studies should also aim to further validate this approach on other datasets and problems to further explore the generalisability of this technique.

## 5. Future Work and Limitations

Although the new model holds potential for drug discovery in the scenarios considered, more research is needed to validate the finding in terms of experimentally using in vitro and eventually in vivo studies. For example, the SMILES structure for tenascin-C was not available and there is currently no easy way to conduct docking for protein-to-protein interactions. For example, the HADDOCK server, whilst potentially useful for protein–protein complex structure predictions, requires user input of known actively interacting residues [50], which is difficult to provide for novel compounds. In addition, these approaches, including, e.g., ClusPro, do not provide affinity estimates but rather less easy-to-interpret scoring methods such as cluster energy scores [51]. MDockPP can provide an intuitive ITScore-PP score that has a correlation of 0.71 in relation to binding affinity [52]. However, this approach is limited in that it takes about a day to generate the results, and further work is also required to improve the correlation to binding affinity. Nonetheless, future studies should aim to further research into approaches for docking across protein-to-protein interactions as well as conducting in vitro analyses to further validate the findings in this study. Future work may also aim to further improve upon existing available approaches such as Convolutional Neural Network based approaches, DeepDTA [27] and MolTrans [28], potentially incorporating knowledge of relationships in the substructural elements across compounds and target on top of the existing sample-level representation here, as part of a multi-scale approach. Toxicity studies have not been conducted and may be required to further understand the implications of the new compounds identified in terms of biological effects in animals and humans. Specifically, the inclusion of toxicity screening as well as absorption, distribution, metabolism, excretion, and toxicity (ADMET) of the newly identified compounds could further increase the clinical relevance of the current findings. Future work should also consider the application of the methodology developed herein to other domains and datasets. For example, it would be interesting to assess the approach here to predict miRNA–lncRNA interactions and disease–cRNA interactions [25,26]. Future work should also assess the interactions among the new antiarrhythmic drugs and the anticoagulant compounds in order to further ascertain how well the compounds fit into existing clinical workflows. In addition, future studies should consider Matthews Correlation Coefficient (MCC) and Balanced Accuracy (BA) metrics, which may also be useful for assessing imbalanced datasets. Future study should also further ascertain whether the regularisation-like effect of contrastive learning can help to mitigate against the correlation effects of similar binders in relation to analogue bias. While pre-trained large models such as ProtBERT are beyond the scope of the current study, future work should assess their potential effect on performance when combined with the methodologies in this study. In terms of thresholding activity (e.g., functional assay) datasets to consider active and inactive categories, this was considered where online functional assay datasets from ChEMBL were considered in relation to the drug compounds and targets. However, it was noted that there was a high level of missingness such that this was challenging to use without supplementation with experimentally assessed activity values, for which such resources were not available in this study. Although additional datasets such as the natural compound dataset have been considered here, future studies should aim to develop better linkage methods across experimental studies to aggregate activity-related datasets through wider collaborations, such as multi-institutional studies. In terms of future directions, it has also been suggested that improved assessment of scenarios for resuming anticoagulants, or alternative left atrial appendage (LAA) occlusion as well as new anticoagulants that inhibit Factor XI, would likely be beneficial [53]. The Factor XI-inhibiting drug abelacimab is currently being evaluated in clinical trials and it may therefore be worth evaluating novel candidate markers against this new drug in future studies of this kind [54].

## 6. Conclusions

The treatment and management of atrial fibrillation pose substantial complexity in terms of the delicate balance in the trade-off between the minimising risk of stroke without increasing the risk of bleeding through anticoagulant optimisations. This study presented a new high-performing model under low-resource settings that identified new natural therapeutic candidates for pharmacological cardioversion and anticoagulation as part of a case study.

## Figures and Tables

**Table 1:** Summary of the BIOSNAP and DUD-E dataset samples included. Training, validation, and test dataset values represent the total number of combinations (number of interaction pairs/number of non-interaction pairs); the DUD-E dataset is only used in the training process; hence, validation and test set boxes are left blank.

Dataset	Drugs	Targets	Training	Validation	Test
**BIOSNAP**	4510	2181	19,238 (9670/9568)	2748 (1396/1352)	5497 (2770/2727)
**DUD-E**	852,292	57	415,204 (8996/406,208)	—	—

**Table 2:** Summary of the NPASS 2018 dataset.

	*N*
**Natural Products**	30,926
**Organisms**	25,041

**Table 3:** Evaluation of the performance of base models without contrastive learning; PR AUC: Precision–Recall Area Under the Curve.

	PR AUC	
	Validation Set	Test Set
**ConPLex**	0.7154	0.7206
**New model**	0.8140	0.8369

**Table 4:** Evaluation of performance of the best-performing model with contrastive learning; PR AUC: Precision–Recall Area Under the Curve.

	PR AUC	
	Validation Set	Test Set
**ConPLex**	0.6999	0.6943
**New model**	0.8118	0.8134

**Table 5:** The interactions between antiarrhythmic drugs with that of the target protein ADRB1.

Euclidean Distance	Compound Name
0.00	ADRB1
5.92	Bisoprolol class 2
6.18	Lidocaine class 1
7.08	Flecainide class 1
8.86	Quinidine class 1
9.64	Disopyramide class 1
10.06	Diltiazem class 4
10.11	Sotalol class 3
10.48	Procainamide class 1
11.27	Mexiletine class 1
12.30	Digoxin-class cardiac glycoside
12.47	Amiodarone class 3
14.03	Verapamil class 4

**Table 6:** The interaction between anticoagulants and Factor X.

Euclidean_Distance	Compound_Name
0.00	factorX
5.98	Apixaban
7.82	Edoxaban
7.92	Dabigatran
9.80	Enoxaparin
14.55	Warfarin
15.01	Rivaroxaban

**Table 7:** The interaction between natural compounds and Factor Xa.

Euclidean Distance	Compound Name
0.00	factorX
0.08	NPC55443
0.10	NPC207866
0.15	NPC194593
0.18	NPC306696
0.18	NPC474341
0.20	NPC62927
0.21	NPC265856
0.25	NPC275027
0.26	NPC195466

## Data Availability

The BIOSNAP dataset is available at: https://snap.stanford.edu/biodata/ (accessed on 27 December 2024) and the DUD-E dataset is available at https://dude.docking.org/targets (accessed on 27 December 2024). The natural compound dataset is available at: https://bidd.group/NPASS/ (accessed on 5 January 2025).

## Supplementary Materials

The following supporting information can be downloaded at: https://www.mdpi.com/article/10.3390/biomedicines13061323/s1, Table S1: The top 10 natural products that interact with ADRB1; Table S2: The interaction of antiarrhythmic drugs with HCN1; Table S3: The top 10 natural products that interact with HCN1; Table S4: Evaluation of computational time and hardware cost performance of base models without contrastive learning (CL) in minutes against comparative ConPLex models here and that with GPU in original Singh et al. [22] paper; PR AUC: Precision-Recall Area Under the Curve; Table S5: Evaluation of performance of base models without contrastive learning; ROC AUC: Area Under the Receiver Operating Characteristic Curve; Table S6: Evaluation of performance of the best performing model with contrastive learning; ROC AUC: Area Under the Receiver Operating Characteristic Curve; Figure S1: Visualisation of the attention weights for the interaction between natural compounds and Factor X. The left and right sides show the attention matrices for each of the cross modal attention heads. The rows represent the samples in the natural compound dataset and columns represent the (p/2 = 512) dimension of the attention head parameter; Figure S2: Using a violin plot, the distribution of interaction scores for Factor X against the clinical anticoagulants (blue) and natural compounds (grey) are shown; *p*-value shows results from one-sided *t* test.
