# Supplementary material for: A Deep Learning Methodology for Screening New Natural Therapeutic Candidates for Pharmacological Cardioversion and Anticoagulation in the Treatment and Management of Atrial Fibrillation
Source: Biomedicines. 2025 May 28;13(6):1323. doi: 10.3390/biomedicines13061323 (PMC12189732; doi:10.3390/biomedicines13061323)
Supplement: Supplementary file 1 [file biomedicines-13-01323-s001.zip › biomedicines-3562053-supplementary.pdf]

Supplementary materials

Table S1. The top 10 natural products that interact with ADRB1.

Euclidean distance	Compound name
0.00	ADRB1
0.10	NPC55443
0.10	NPC265856
0.14	NPC234730
0.17	NPC474341
0.18	NPC207866
0.20	NPC306696
0.25	NPC477413
0.26	NPC121649
0.27	NPC194593

Table S2. The interaction of antiarrhythmic drugs with HCN1.

Euclidean distance	Compound name
0.00	HCN1
4.95	Bisoprolol class 2
5.80	Lidocaine class 1
8.13	Flecainide class 1
10.12	Sotalol class 3
11.30	Quinidine class 1
12.45	Mexiletine class 1
12.85	Digoxin class Cardiac glycoside
14.55	Diltiazem class 4
14.83	Procainamide class 1
15.56	Disopyramide class 1
16.86	Amiodarone_class 3
18.27	Verapamil class 4

Table S3. The top 10 natural products that interact with HCN1.

Euclidean distance	Compound name
0.00	HCN1
0.04	NPC262615
0.12	NPC118813
0.13	NPC326241
0.15	NPC197357
0.18	NPC193870
0.23	NPC321536
0.23	NPC19631
0.23	NPC194411
0.23	NPC329401

Table S4. Evaluation of computational time and hardware cost performance of base models without contrastive learning (CL) in minutes against comparative ConPLex models here and that with GPU in original Singh et al[20] paper; PR AUC: Precision-Recall Area Under the Curve.

	Time (min)		Hardware configuration	Hardware cost
	without CL	with CL		
**ConPLex**	8.6	69.4	14 inch MacBook Pro with Apple M1 Pro chip and 16 GB memory	Approx. £1,599.00
**New model**	32.3	165.8		
**Singh et al (ConPLex With GPU)[20]**	NA	1273	112-core Intel Xeon Gold 6258R CPU and NVIDIA A100 GPU	Approx. £13,712 + £13,156.02 = £26,868

Table S5. Evaluation of performance of base models without contrastive learning; ROC AUC: Area Under the Receiver Operating Characteristic Curve.

	ROC AUC	
	Validation set	Test set
**ConPLex**	0.6620	0.6947
**New model**	0.8004	0.8037

Table S6. Evaluation of performance of the best performing model with contrastive learning; ROC AUC: Area Under the Receiver Operating Characteristic Curve.

	ROC AUC	
	Validation set	Test set
**ConPLex**	0.6776	0.6770
**New model**	0.7926	0.7825

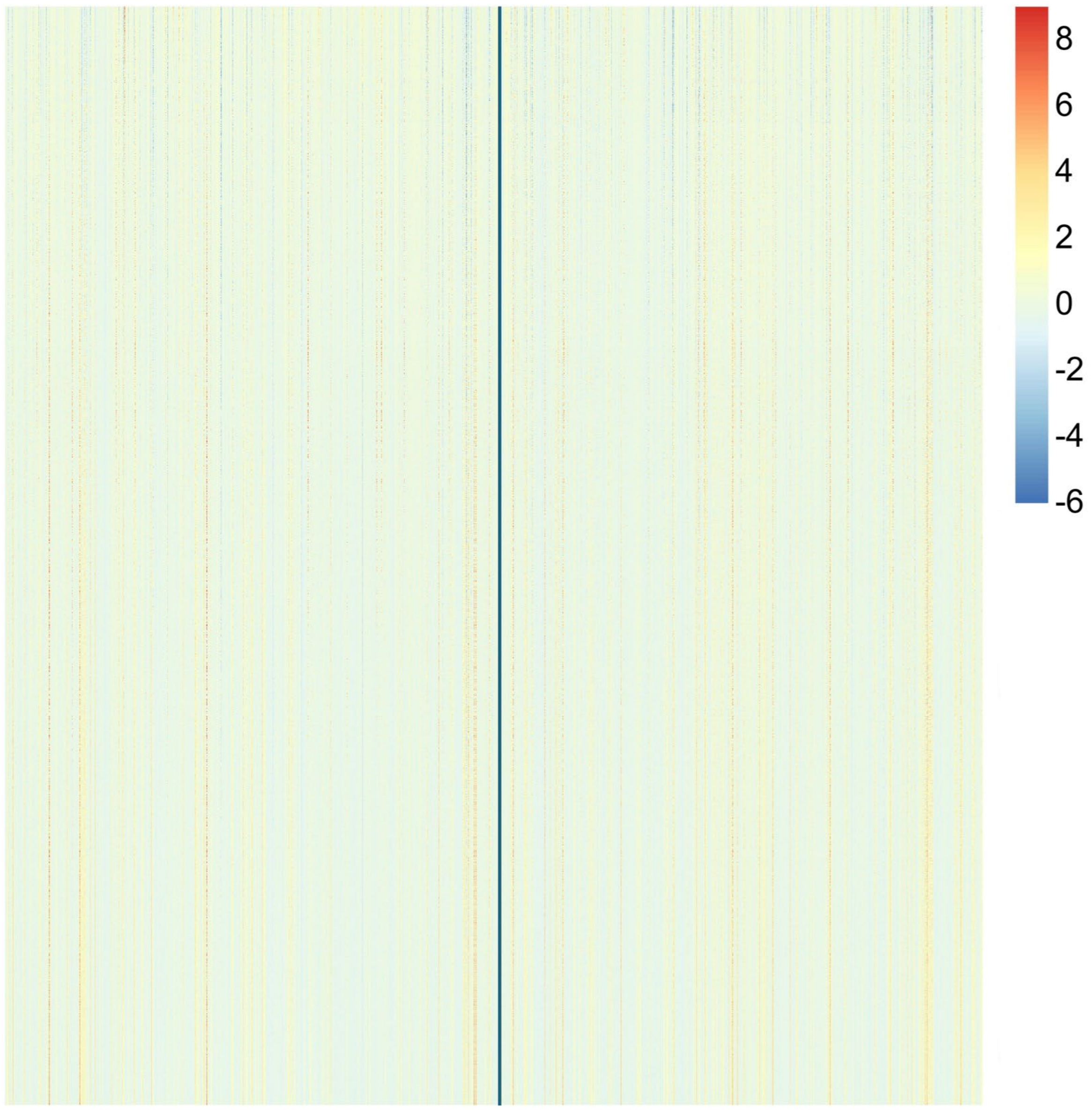

Figure S1. *Visualisation of the attention weights for the interaction between natural compounds and Factor X. The left and right sides show the attention matrices for each of the cross modal attention heads. The rows represent the samples in the natural compound dataset and columns represent the* $(p/2 = 512)$ *dimension of the attention head parameter.*

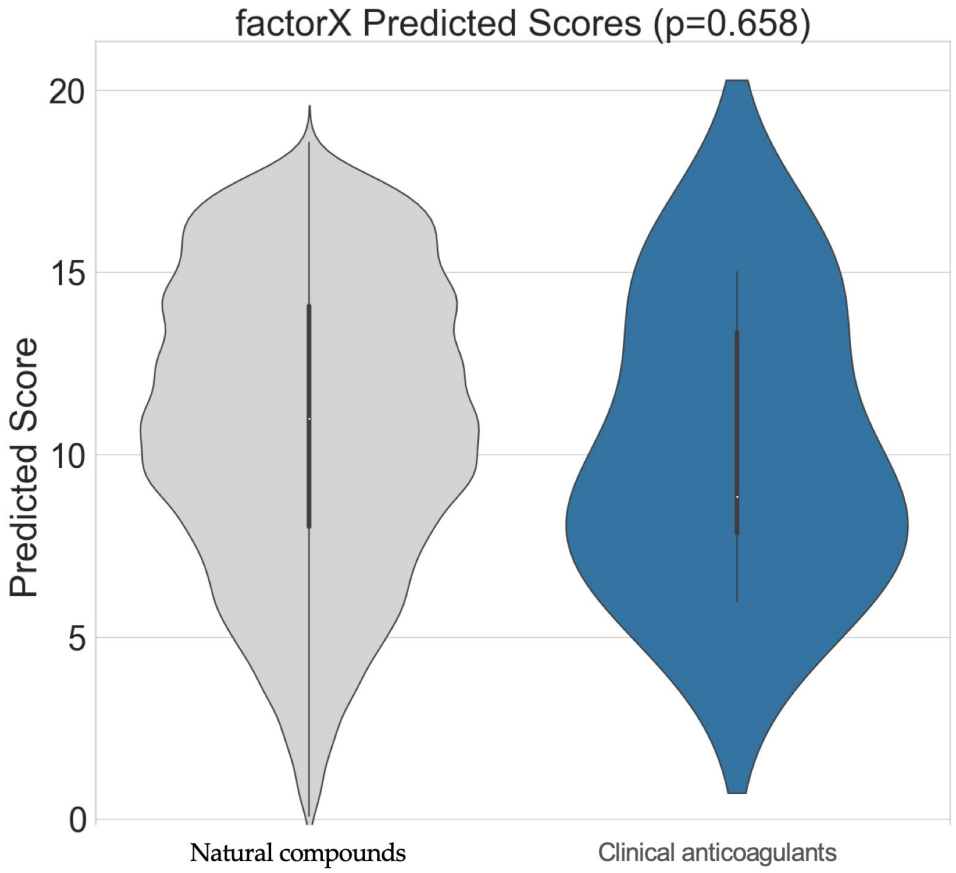

Figure S2. Using a violin plot, the distribution of interaction scores for Factor X against the clinical anticoagulants (blue) and natural compounds (grey) are shown; P-value shows results from one-sided t test.
